# Phlebotomine mortality effect of systemic insecticides administered to dogs

**DOI:** 10.1186/s13071-018-2820-x

**Published:** 2018-04-05

**Authors:** Sonia Ares Gomez, Javier Lucientes Curdi, Juan Antonio Castillo Hernandez, Paz Peris Peris, Adriana Esteban Gil, Ronald Vladimir Oropeza Velasquez, Paula Ortega Hernandez, Albert Picado

**Affiliations:** 1 ISGlobal, Barcelona Ctr. Int. Health Res. (CRESIB), Hospital Clínic-Universitat de Barcelona, Barcelona, Spain; 2 Department of Animal Pathology, Faculty of Veterinary Medicine at the University of Zaragoza, Zaragoza, Spain

**Keywords:** Zoonotic visceral leishmaniasis (ZVL), Sand flies, Dogs, Systemic insecticides

## Abstract

**Background:**

Zoonotic visceral leishmaniasis (ZVL) caused by *Leishmania* (*Leishmania*) *infantum* is an important disease in humans and dogs. Different mammal species are reservoirs but dogs are considered to be the main one. Phlebotomine sand flies are the proven vector. Four systemic insecticides approved for their use in dogs were previously selected based on their potential to be used in endemic countries as part of the control programs of ZVL. These insecticides are proved to be safe and effective against the on-label insects and parasites, but there is no information about their activity against phlebotomine sand flies.

**Methods:**

The phlebotomine mortality of four systemic insecticides in dogs was evaluated using two randomized clinical trials. For the first trial, thirty dogs were randomly allocated into five groups: four treatments and one control, of equal size. The treatments evaluated were: Guardian®SR, Elanco (moxidectin); Comfortis®, Elanco (spinosad); Bravecto®, Merck Animal Health (fluralaner); and NexGard®, Merial (afoxolaner). Blood from dogs was taken at days 2, 4, 21 and 31 post-treatment (trial 1). The compound that showed the highest efficacy was selected for a second trial (trial 2) with 20 dogs sampled at days 0, 2, 4, 7, 14, 18, 32, 39, 51 and 84 post-treatment. Membrane feeding bioassays with *Phlebotomus papatasi* were used to evaluate the phlebotomine mortality efficacy of the different treatments. Phlebotomine mortality was observed every 24 h following the membrane feeding during 5 days. A mixed model for a negative binomial logistic regression, and a Cox proportional hazard mixed model were used to estimate phlebotomine mortality due to different treatments.

**Results:**

Fluralaner was the only compound that showed significant phlebotomine mortality. Fluralaner maintained the phlebotomine mortality between 60–80% for 30 days after treatment. In trial 1 we found that fluralaner increased the risk of death by 1.9 times (95% CI: 1.02–3.6) and 1.7 times (95% CI: 1.09–2.6) at days 2 and 4 after treatment. The Cox model resulted in an increase of 1.47 (95% CI: 1.1–1.96) times in hazard risk at day 2 and 1.89 (95% CI: 1.35–2.45) at day 4 after treatment.

In trial 2 we found that fluralaner increased the risk of death by 1.64 times (95% CI: 1.16–2.54) and 1.97 times (95% CI: 1.23–3.17) at days 14 and 32. The hazard risk was also increased by 1.92 (95% CI: 1.4–2.64) times at day 14 after treatment. Phlebotomine survival including all experimental days was significantly lower in the fluralaner group in both trials.

**Conclusions:**

A single oral treatment of fluralaner in dogs induces phlebotomine mortality. Systemic insecticides in dogs should be considered as a potential preventive measure of ZVL.

**Electronic supplementary material:**

The online version of this article (10.1186/s13071-018-2820-x) contains supplementary material, which is available to authorized users.

## Background

Zoonotic visceral leishmaniasis (ZVL) is a public health concern in some regions (e.g. Brazil, Mediterranean basin, Iran and China) where it causes a significant number of human cases, particularly in children [1]. The disease can be lethal if appropriate treatment is not provided [2]. The causal agent is a protozoan parasite named *Leishmania* (*Leishmania*) *infantum*. Different mammal species are reservoirs but dogs are considered to be the main one [3] and phlebotomine sand flies are the proven vector [4]. Current control measures (e.g. culling of infected dogs, insecticide spraying, dog collars) have a limited impact on ZVL transmission for several reasons [5]. New cost-effective vector control methods are required. Systemic insecticides that are easily administered to dogs (e.g. orally) and kill sand flies biting on them for a significant amount of time, could be used as an alternative or complement to control ZVL in endemic regions [6].

A number of systemic insecticides for dogs are currently available. These are mainly registered to treat and prevent ectoparasitic infestations such as fleas, ticks, and mites [7–9]. Others are used to prevent dirofilarial infections in dogs [10]. Systemic insecticides enter into the circulatory system to be distributed through the entire body. The route of administration can be oral, parenteral, or topical [11].

The compounds considered as systemic insecticides currently used in dogs are isoxazolines (fluralaner, afoxoloner, sarolaner), spinosyns (spinosad), neonicotinoids (nitenpyram), and macrocyclic lactones (ivermectin, selamectin, moxidectin, and doramectin). Isoxazolines are antagonist of the gamma-aminobutyric acid (GABA)-gated chloride channels. Several studies have demonstrated the isoxazolines efficacy against fleas and ticks infestations after a single oral administration [12–14]. The duration of the effect ranges from four (afoxoloner, sarolaner) to 12 weeks (fluralaner). Macrocyclic lactones act as GABA agonists and agonists of the glutamate-gated chloride channels. Systemically distributed macrocylic lactones are used to control heartworm infections in dogs (*Dirofilaria immitis*) [15].

Some of these insecticides have phlebotomine mortality effect as shown in experimental [16, 17] and field studies [18]. There is, however, no evidence of their effect on sand flies when administered to dogs. In this study we evaluate the phlebotomine mortality effect post-feeding in blood of dogs treated with four systemic insecticides: moxidectin, spinosad, fluralaner and afoxolaner.

## Methods

### Systemic insecticides

The insecticides evaluated in this study were selected based on their: (i) phlebotomine mortality showed in laboratory experiments or a potential phlebotomine mortality based on the insecticide mechanism of action; (ii) optimal plasma concentration for the phlebotomine mortality effect based on *in vivo* or *in vitro* studies; (iii) long-lasting action (> 12 weeks); (iv) ease of administration (e.g. oral); and (v) availability as commercial products for dogs [6]. The four compounds evaluated were: Guardian®SR, Elanco (moxidectin); Comfortis®, Elanco (spinosad); Bravecto®, Merck Animal Health (fluralaner); and NexGard®, Merial (afoxolaner). All the products where administered just once and following the label indications: Guardian®SR, subcutaneous injection dose of 0.17 mg/kg body weight; Comfortis®, chewable table 425 mg for dogs > 6–9.4 kg; Bravecto®, chewable tablet 500 mg for dogs > 4.5–10 kg, and NexGard®, chewable tablet 28.3 mg for dogs > 4–10 kg. Fluralaner was administered on fasted dogs in trial 1 and on fed dogs in trial 2 as it has shown to increase bioavailability [19].

### Study design

The study was run between February 2016 and June 2017. Two randomized controlled trials were conducted in dogs. The first clinical trial (trial 1) was designed to evaluate the effect of four systemic insecticides (see above). The compound showing the best performance in trial 1 was further evaluated in the second clinical trial (trial 2). Trial 2 was designed to measure the phlebotomine mortality of the selected compounds for three months. The phlebotomine mortality of the systemic insecticides was measured by comparing the mortality of sand flies feeding on blood from treated and control dogs.

#### Study site and dogs

The study was conducted in one breeding kennel located in the Province of Huesca (Spain). Dogs from 1–5 years-old, from the breeds Parson Rusell (trial 1: *n* = 7; trial 2: *n* = 11), Jack Rusell (trial 1: *n* = 13; trial 2: *n* = 9) and Teckel (trail 1: *n* = 10) participated in the study. To avoid contamination, no insecticide treatment was provided to the dogs or the kennel in the three months preceding the study. All of the dogs each had a microchip and were examined by a veterinarian at the start of the study. Only healthy dogs were enrolled. The enrolled dogs were kept in the kennel with their regular routines and diet for the whole study period.

#### Sample size

The sample size calculation in trial 1 aimed at detecting at least a 65% increase in phlebotomine mortality at 24 h after feeding on a treated dog’s blood collected up to 30 days post-treatment. Trial 2 was powered to detect at least 45% increase in phlebotomine mortality 24 h after feeding on a treated dog’s blood up to 90 days post-treatment. These calculations were done assuming 20% phlebotomine mortality in control group, alpha error of 0.05 and 80% power [20]. The sample size calculation resulted in 6 dogs per treatment group in trial 1 (24 dogs in 4 treatment groups of equal size and 6 dogs in control) and 10 dogs per treatment group in trial 2 (10 dogs in treatment and 10 dogs in control).

#### Randomization and allocation

We selected dogs (trial 1: *n* = 30; trial 2: *n* = 20) of similar size from the kennel that participated in the study. Once the dogs were selected the randomization was done using the dogs’ microchip numbers. To achieve balance across treatment groups a stratified permuted block randomization was used [21]. Dogs were subdivide into strata (breed and gender) then a permuted block randomization was used for each stratum [22]. The permuted block randomization was performed using the sample function in R (version 3.3) [23]. The only person aware of the randomization results was the researcher who administered the treatments. Researchers responsible for taking the blood samples and performing the membrane feedings were blind to the treatment allocation.

Treatments in the intervention groups were administered in a unique dose at Day 0 following the label indications. Comfortis®, Bravecto®, and NexGard® were given orally and Guardian® SR was administered subcutaneously. The control dogs received no treatment.

Blood samples from the cephalic or jugular veins were taken on days 2, 4, 21 and 31 post-treatment in trial 1 and on days 0, 2, 4, 7, 14, 18, 32, 39, 51 and 84 in trial 2 (Additional file 1: Figure S1). At each time-point 4 ml of blood per dog was kept in heparinized tubes preserved at -21 °C for 3–9 months until the bioassays were performed. Blood samples were identified with the date of collection and the dog’s microchip number.

#### Outcomes

The effect of the four systemic insecticides was measured by membrane feeding bioassays using reared *Phlebotomus papatasi* sand flies from the School of Veterinary Medicine at the University of Zaragoza. To summarize, the colony was maintained at 30 °C, 90% relative humidity, and a photo period of 17:7 h (light:dark photocycle). The sand flies were deprived of sucrose solution 12 h before the bioassays. The dog’s blood was thawed at room temperature (23 °C) on the day of the bioassay. Two- to seven-days-old *P. papatasi* sand flies were exposed to the blood collected from the dogs in the study using Hemotek (Discovery Workshops, Lancashire, UK [24]). Hemotek kept the blood at 37 °C and chicken skin membrane was used to mimic blood-feeding on-animal conditions.

The membrane feeding bioassays for each blood sample were performed with around 40 females of *P. papatasi* mixed with some males. The sand flies were allowed to feed on the dog’s blood for at least 1.5 h or until 15 females were observed to be fully engorged; a total of 1800 fully engorged sand flies were needed for trial 1 and 3000 for trial 2.

After the membrane feeding, the 15 fully engorged sand flies were placed into individual plastic cups of 90 ml and 60 mm diameter. An orifice of 45 mm was made in the bottom of the cup and covered with clay to keep humidity high inside the cup. The selection of the fully engorged sand flies was made by visual observation. Sucrose solution was provided to all sand flies. Phlebotomine mortality was recorded every 24 h for five days.

### Statistical analysis

The average mortality and its 95% confidence interval (CI) of the 15 fully engorged sand flies were calculated per treatment group (control, afoxolaner, flularaner, moxidectin and spinosad), sampling day (trial 1: 2, 4, 21 and 31; and trial 2: days 0, 2, 4, 7, 14, 18, 32, 39, 51 and 84) and mortality recording time (24, 48, 72, 96 and 120 h).

Two different statistical analyses were used to assess the anti-phlebotomine mortality of systemic insecticides. First, a mixed model for a negative binomial logistic regression [25] was performed to estimate differences in phlebotomine mortality 24 h after blood ingestion between treatment and control groups over time (30 days in trial 1 and 60 days in trial 2). The model analyzed the incidence rate (IR) as the response variable, that is, the number of fully engorged sand flies dead in 24 h given the total number of fully engorged sand flies (offset). The exponential of the model estimates are the incidence rate ratios (IRR) comparing each treatment to control. The variable dog was included as a random effect to account for the repeated sampling. Sampling day, treatment, and their interaction were the explanatory variables. The interaction term was included to be able to capture differences among the treatments in their efficacy over time (e.g. one treatment could have the highest effect 2 days after administration but another treatment could have the highest effect 4 days after administration). Secondly, survival analyses were conducted. Kaplan Meier curves were used to represent the phlebotomine mortality up to 5 days post-feeding for treatment and control groups. Then, a Cox proportional hazard mixed model for the number of dead sand flies was used to estimate the mean increase in the risk of phlebotomine mortality per day in treated groups compared to control. The Cox proportional hazard model response variable is the effect of treatment in the hazard rate of a phlebotomine dying (i.e. probability of death in 24 h) after feeding on a dog’s blood. The exponential of the model estimates are the hazard rate ratios (HRR) comparing each treatment to control. As for the negative binomial model, dog was included as a random effect and sampling day, treatment, and their interaction were the explanatory variables. The z-statistic was used to determine statistical significance of the estimates obtained from both models. Bayesian information criterion (BIC) was used to select the best models.

All the analyses were conducted in R (version 3.3) [23]. The *MASS* package [26] was used to conduct de negative binomial regression. The *survival* and *coxme* packages [27, 28] were used to conduct the Kaplan Meier curves and the Cox proportional hazard mixed model respectively.

## Results

### Trial 1

Six dogs per group were included in trial 1. Gender and breed were evenly distributed among groups (Additional file 2: Table S1). No adverse effects related to the administration of the treatments were observed in any dog.

The total number of fully engorged sand flies evaluated was 1700. The total number of sand flies used per treatment groups was 360, 316, 365, 330 and 329 for control, afoxolaner, fluralaner, moxidectin and spinosad, respectively. Phlebotomine mortality, the corresponding percentage and its 95% CI from the mortality observed at 24, 48, 72, 96 and 120 h after blood-feeding for treatment and control groups and sampling day are reported in Additional file 2: Table S2.

**Fig. 1:**
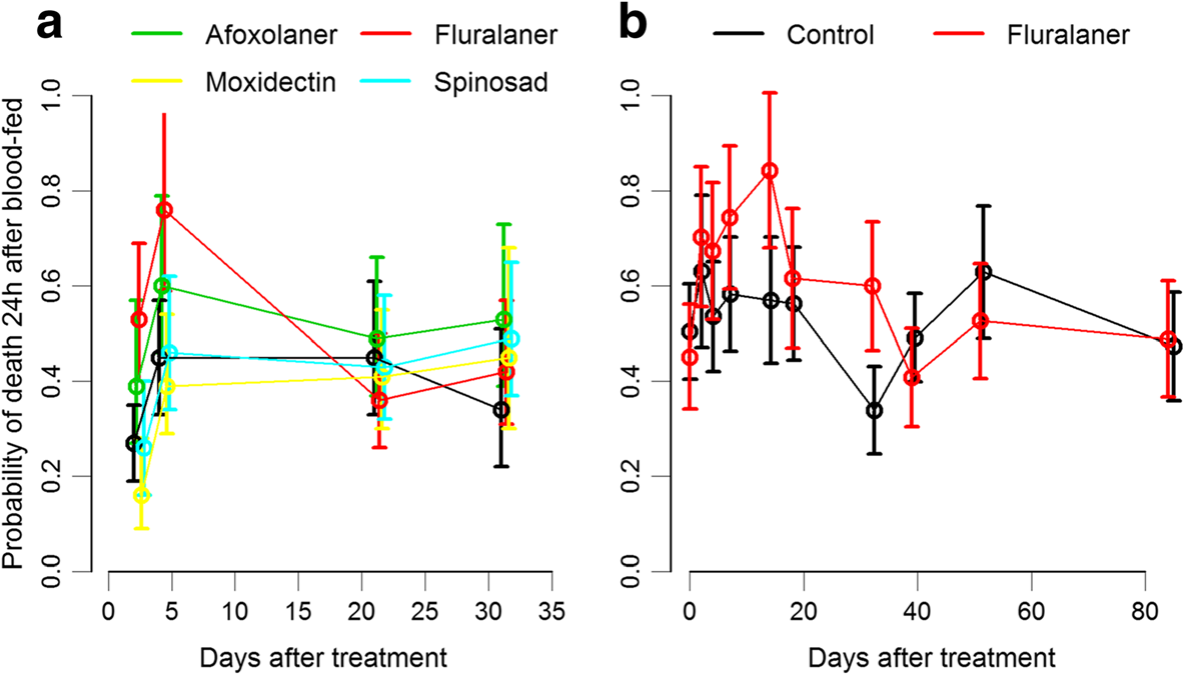
Probability of death for a phlebotomine 24 h after blood-feeding for each treatment group and sampling day estimated from a mixed model for a negative binomial logistic regression. The variable dog was included as a random effect, treatement, days after treatment, and their interaction were covariates. **a** Trial 1: afoxolaner *vs* fluralaner *vs* moxidectin *vs* spinosad *vs* control. **b** Trial 2: fluralaner *vs* control

Compared to control, phlebotomine mortality 24 h post-feeding was higher in the sand flie group fed in blood samples of dogs treated with fluralaner at days 2 (55%; 95% CI: 45–65%) and 4 (77%; 95% CI: 68–86%) post-insecticide-treatment. The phlebotomine mortality for the fluralaner group was similar to the control group at days 21 (37%; 95% CI: 27–47%) and 31 (43%; 95% CI: 32–53%) post-treatment. No differences were observed in the phlebotomine mortality in the other treatments. The negative binomial mixed model confirmed the previous observations. Treating dogs with fluralaner increased the risk of death for sand flies by 1.9 (95% CI: 1.02–3.6) and 1.7 (95% CI: 1.09–2.6) times at day 2 and 4, respectively, compared to untreated dogs. None of the treatments had an effect on phlebotomine mortality at days 21 and 31 (Table 1 and Fig. 1a).

**Table 1:** Trial 1 results from the negative binomial mixed model that included treatment group, sample day and their interaction as fixed effects and dog as random effect. Results are presented as the incidence rate ratio (IRR) which is the increase in the rate of incidence compared with control at each sampling day

Covariates effect^a^	IRR^b^	95% CI	*P*-value
Day 2: Control	Reference	–	–
Afoxolaner	1.39	0.74–2.67	0.31
Fluralaner	1.94	1.14–3.39	0.01*
Moxidectin	0.60	0.26–1.29	0.21
Spinosad	0.90	0.43–1.80	0.77
Day 4: Control	Reference	–	–
Afoxolaner	1.34	0.82–2.18	0.23
Fluralaner	1.68	1.05–2.71	0.03*
Moxidectin	0.77	0.45–1.31	0.34
Spinosad	1.02	0.61–1.70	0.92
Day 21: Control	Reference	–	–
Afoxolaner	1.11	0.67–1.89	0.67
Fluralaner	0.81	0.47–1.38	0.45
Moxidectin	0.91	0.54–1.53	0.73
Spinosad	0.96	0.57–1.61	0.89
Day 31: Control	Reference	–	–
Afoxolaner	1.51	0.81–2.89	0.20
Fluralaner	1.21	0.64–2.31	0.55
Moxidectin	1.33	0.65–2.74	0.41
Spinosad	1.41	0.77–2.66	0.27

^a^The covariate effects used control group at each corresponding day after treatment as baseline comparison [Pr(>|z|)]

^b^Incidence rate ratio, as the ratio between the incidence rate in control group and the incidence rate for each treatment group, at the corresponding sampling day after treatment

^*^Significance level defined at α = 0.05

**Fig. 2:**
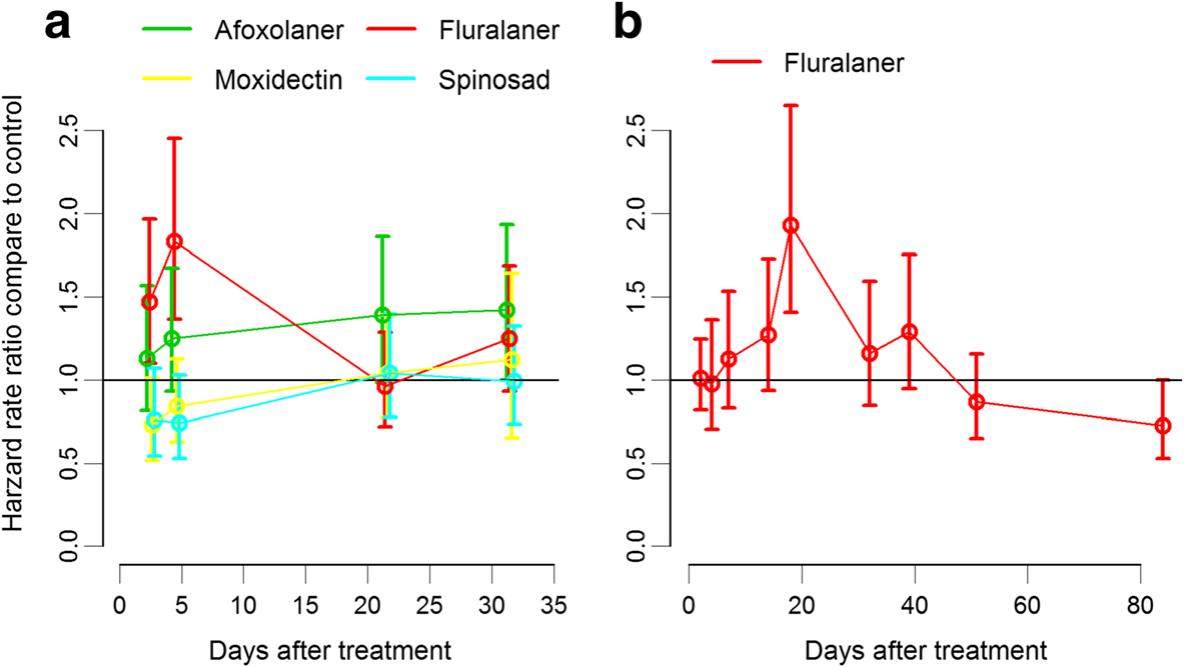
Hazard rate ratio compared with control for each treatment group and sampling day estimated from a mixed cox proportional hazard model. The variable dog was included as a random effect treatment, days after treatment, and their interaction were covariates. **a** Trial 1: afoxolaner *vs* fluralaner *vs* moxidectin *vs* spinosad *vs* control. **b** Trial 2: fluralaner *vs* control

Kaplan Meier (KM) curves also indicated that fluranaler was the only compound that had a phlebotomine mortality effect. At days 2 and 4 the KM curves showed the biggest difference in phlebotomine survival comparing fluralaner with control at 24 and 48 h after blood-feeding. At 80 h after blood-feeding, phlebotomine survival is between 0 and 20% with no differences among days or treatment groups (Additional file 3: Figure S2). These observations were confirmed by the cox proportional hazard model that showed significant increase in hazard risk (HR) only for the fluralaner group on days 2 and 4 after treatment (Additional file 4: Figure S3). The hazard risk in fluralaner treated dogs was 1.47 (95% CI: 1.1–1.96) times higher than control at day 2 after treatment. This hazard increased to 1.89 (95% CI: 1.35–2.45) at day 4 after treatment (Table 2). However, at days 21 and 31 after treatment the effect of fluralaner decreases and the hazard ratio is no longer significantly above 1 (Table 2 and Fig. 2a).

**Table 2:** Trial 1 results from the cox proportional hazard model that included treatment group, sample day and their interaction as fixed effects and dog as random effect. Results are presented as the hazard rate ratio (HRR) which is the increase in the hazard rate compared with control at each sampling day

Covariates effect^a^	HRR^b^	95% CI	*P*-value
Day 2: Control	Reference	–	–
Afoxolaner	1.13	0.85–1.56	0.45
Fluralaner	1.47	1.10–1.96	< 0.001*
Moxidectin	0.73	0.51–1.01	0.06
Spinosad	0.76	0.54–1.08	0.08
Day 4: Control	Reference	–	–
Afoxolaner	1.25	0.93–1.67	0.08
Fluralaner	1.83	1.36–2.45	< 0.001*
Moxidectin	0.84	0.62–1.13	0.17
Spinosad	0.74	0.53–1.02	0.33
Day 21: Control	Reference	–	–
Afoxolaner	1.39	0.98–1.86	0.07
Fluralaner	0.96	0.72–1.28	0.91
Moxidectin	1.06	0.78–1.39	0.55
Spinosad	1.04	0.76–1.38	0.61
Day 31: Control	Reference	–	–
Afoxolaner	1.42	0.99–1.93	0.08
Fluralaner	1.25	0.93–1.68	0.43
Moxidectin	1.12	0.65–1.64	0.57
Spinosad	0.99	0.73–1.32	0.37

^a^The covariate effects used control group at each corresponding day after treatment as baseline comparison [Pr(>|z|)]

^b^Hazard rate ratio, as the ratio between the hazard rate in control group and the hazard rate for each treatment group, at the corresponding sampling day after treatment

^*^Significance level defined at α = 0.05

### Trial 2

Ten dogs per group were included in trial 2. Gender and breed were evenly distributed among groups (Additional file 2: Table S1). No adverse effects related to fluranaler administration were observed.

The total number of fully engorged sand flies evaluated was 2982: 1347 for fluralaner and 1635 for the control group. Phlebotomine mortality, the corresponding percentage and its 95% CI from the mortality observed at 24, 48, 72, 96 and 120 h after blood-feeding for treatment and control groups and sampling day are reported in Additional file 2: Table S3.

Compared to the control, phlebotomine mortality 24 h after blood-feeding was higher for the fluralaner group at days 14 (85%; 95% CI: 78–90%) and 32 (60%; 95% CI: 52–68%) after treatment (Additional file 4: Figure S3). The negative binomial mixed model confirmed that treating dogs with fluralaner increased the risk of death for sand flies by 1.64 (95% CI: 1.16–2.54) and 1.97 (95% CI: 1.23–3.17) times at day 14 and 32 after treatment respectively (Table 3 and Fig. 1b).

**Table 3:** Trial 2 results from the negative binomial mixed model that included treatment group, sample day and their interaction as fixed effects and dog as random effect. Results are presented as the incidence rate ratio (IRR) which is the increase in the rate of incidence compared with control at each sampling day

Covariates effect^a^	IRR^b^	95% CI	*P*-value
Day 2: Control	Reference	–	–
Fluralaner	1.24	0.78–1.96	0.35
Day 4: Control	Reference	–	–
Fluralaner	1.40	0.90–2.18	0.13
Day 7: Control	Reference	–	–
Fluralaner	1.42	0.92–2.19	0.11
Day 14: Control	Reference	–	–
Fluralaner	1.64	1.16–2.54	0.025*
Day 18: Control	Reference	–	–
Fluralaner	1.22	0.77–1.91	0.38
Day 32: Control	Reference	–	–
Fluralaner	1.97	1.23–3.17	0.004*
Day 39: Control	Reference	–	–
Fluralaner	0.92	0.59–1.44	0.73
Day 51: Control	Reference	–	–
Fluralaner	1.24	0.93–1.66	0.14
Day 84: Control	Reference	–	–
Fluralaner	0.93	0.68–1.27	0.68

^a^The covariate effects used control group at each corresponding day after treatment as baseline comparison [Pr(>|z|)]

^b^Incidence rate ratio, as the ratio between the incidence rate in control group and the incidence rate for each treatment group, at the corresponding sampling day after treatment

^*^Significance level defined at α = 0.05

Kaplan Meier (KM) curves also indicated that fluralaner had phlebotomine mortality. For each day of the trial the KM curve showed the biggest difference in phlebotomine survival at 24 h after blood-feeding. At 80 h after blood-feeding phlebotomine survival is between 0 and 10% with no differences between fluralaner and control (Additional file 5: Figure S4). The cox proportional hazard model showed significant increase in hazard risk (HR) only on day 14 after treatment. The hazard risk in fluralaner treated dogs was 1.92 (95% CI: 1.4–2.64) times higher than control at 14 days after treatment (Table 4 and Fig. 2b).

**Table 4:** Trial 2 results from the Cox proportional hazard model that included treatment group, sample day and their interaction as fixed effects and dog as random effect. Results are presented as the hazard rate ratio (HRR) which is the increase in the hazard rate compared with control at each sampling day

Covariates effect^a^	HRR^b^	95% CI	*P*-value
Day 2: Control	Reference	–	–
Fluralaner	0.97	0.70–1.36	0.90
Day 4: Control	Reference	–	–
Fluralaner	1.12	0.70–1.53	0.43
Day 7: Control	Reference	–	–
Fluralaner	1.27	0.94–1.72	0.12
Day 14: Control	Reference	–	–
Fluralaner	1.92	1.40–2.64	<0.0001*
Day 18: Control	Reference	–	–
Fluralaner	1.15	0.84–1.59	0.36
Day 32: Control	Reference	–	–
Fluralaner	1.28	0.94–1.75	0.10
Day 39: Control	Reference	–	–
Fluralaner	0.86	0.65–1.15	0.33
Day 51: Control	Reference	–	–
Fluralaner	0.86	0.64–1.15	0.22
Day 84: Control	Reference	–	–
Fluralaner	0.73	0.53–1.05	0.09

^a^The covariate effects used control group at each corresponding day after treatment as baseline comparison [Pr(>|z|)]

^b^Hazard rate ratio, as the ratio between the hazard rate in control group and the hazard rate for each treatment group, at the corresponding sampling day after treatment

^*^Significance level defined at α = 0.05

The mixed models of both trials were run with dog as random effect. However, this random effect was significant by likelihood ratio test (chi-square test; trial 1, *df *= 29 *P* = 4. 5609e-10; trial 2, *df* = 19, *P* = 6.627005e-12) only for the Cox proportional hazard models.

## Discussion

A single dose of Bravecto® chewable tablet (25 mg/kg of fluralaner) administered to dogs showed a phlebotomine mortality effect. The phlebotomine mortality ranged from 60 to 80% up to 30 days after treatment. This mortality was lower than the mortality reported in fleas. In fleas, the main the target of the product, the mortality reported was 100% 24 hours after infestation during 12 weeks after treatment [12, 29, 30]. The lower mortality effect found in phlebotomine sand flies comparing with the mortality found in flea studies, besides the differences in the study design [direct feeding (fleas) *versus* membrane feeding], could be due to their differences in feeding behavior. For example, sand flies ingest 1 μl of blood or less [31] while fleas ingest 110 μl [32] of blood, sand flies take an average of six minutes to feed on blood [33] while fleas take an average of 25 minutes [34]. These feeding characteristics could influence the amount and the bioavailability of the insecticide.

The other compounds tested: spinosad, afoxolaner, and moxidectin did not show significant increase in phlebotomine mortality. This may be due in part to the low concentration used in this study. For example, we administered spinosad at 45–70 mg/kg; however, previous studies in rodents showed that spinosad treated diet had phlebotomine mortality when administered at 5000 mg/kg but no significant mortality was observed at 500 mg/kg of spinosad [35]. Nevertheless, spinosad, as well as afoxolaner and fluralaner administered at label concentration showed to have significant lethal effects on triatomines directly feeding on dogs [36]. Fluralaner and afoxolaner killed 100% of the triatomines for 51 days after oral administration. Lower mortality (50–70%) was observed in the spinosad group.

Furalaner showed a significant increase in sand fly mortality in both trials 1 and 2. However, the two trials differ in the onset of action and the duration of it. These differences can be explained by (i) higher phlebotomine mortality in control group in trial 2 (phlebotomine mortality at day 2: 63%, CI 53,72) than in trial 1 (day 2: 28%, CI 19, 38), and (ii) the influence of food on the pharmacokinetics (PK) of fluralaner. More precisely, for the first trial, the chewable tablet of fluralaner was administered early in the morning before feeding the dogs: the dogs’ last meal was about 20 hours before administration. In trial 2, the chewable tablet was administered after the dogs received half of their daily food ration. One PK study showed that when fluralaner is administered with food, its bioavailability is duplicated [20].

The mortality associated to the different treatments observed in this study may be an underestimation. Mortality in the control group 24 hours after blood-feeding was much higher (50%, Additional file 4: Figure S3) than expected (20%) and variable (e.g. phlebotomine mortality in trial 2 range from 34% in day 32 to 63% in day 2). The high mortality reduced the power of the trials and the variability may explain the inconsistencies on the effect observed on consecutive sampling days (e.g, statically significant mortality on day 14 and 32 but not on day 21 in trial 2). Despite the high mortality in the control group we demonstrated that systemic insecticides, such as fluralaner have mortality effect against phlebotomine sand flies. Variations of mortality in controls may be due to (i) use membrane feeding instead of direct feeding; (ii) low number of sand flies per membrane feeding; or (iii) phlebotomine environmental temperature or humidity. Reducing mortality in control group may allow better estimation of mortality due to systemic insecticides.

## Conclusions

Despite the limitations, this study shows that a single oral treatment of fluralaner in dogs induces phlebotomine mortality. This would indicate that administering systemic insecticides to dogs could potentially be used to control ZVL, but further work needs to be conducted to define the requirements of compounds to be used in a public health intervention, e.g. insecticide efficacy, duration of the efficacy, route of administration or the percentage of dogs treated in the community. Finally, a clinical trial should be conducted to evaluate the impact of administering systemic insecticides to dogs to control ZVL in humans in endemic areas.

## Additional files

Additional file 1 **Figure S1.** Organization chart showing the sampling schedule. (TIFF 108 kb)

Additional file 2: **Table S1.** Randomization results. **Table S2**. Trial 1, sand fly mortality, percentage, and 95% CI at 24, 48, 72, 96 and 120 hours after blood feeding for treatment group and sampling day. **Table S3**. Trial 2, sand fly mortality, percentage, and its 95% CI at 24, 48, 72, 96 and 120 hours after blood feeding for treatment group and sampling day. **Table S4**. Trial 1 results from the negative binomial mixed model with treatment group, sample day and their interaction as explanatory variables and dog as random effect. **Table S5.** Trial 1 Cox proportional hazard mixed model with treatment group, sample day and their interaction as explanatory variables and dog as random effect. **Table S6**. Trial 2 negative binomial mixed model with treatment group, sample day and their interaction as explanatory variables and dog as random effect. **Table S7.** Trial 2 Cox proportional hazard mixed model with treatment group, sample day and their interaction as explanatory variables and dog as random effect (DOCX 53 kb)

Additional file 3 **Figure S2.** Trial 1, sand fly survival after feeding with blood collected from dogs 2, 4, 21 and 31 days after treatment administration. Groups: control (black); afoxolaner (green); fluralaner (red); moxidectin (yellow); and spinosad (blue). (TIFF 66 kb)

Additional file 4 **Figure S3.** Sand fly mortality percentage and its 95% CI at 24 hours after blood feeding for each treatment group and sampling day. A: Trial 1: afoxolaner *vs* fluralaner *vs* moxidectin *vs* spinosad *vs* control. B: Trial 2: fluralaner *vs* control (TIFF 65 kb)

Additional file 5 **Figure S4.** Trial 2, sand fly survival after feeding with blood collected from dogs 0, 2, 4 ,7 14, 18, 32, 39, 51 and 84 days after treatment administration by treatment groups: control (black), fluralaner (red). (TIFF 37 kb)

## Abbreviations

GABA: Gamma-aminobutyric acid
HRR: Hazard rate ratios
IRR: Incidence rate ratio
PK: Pharmacokinetics
ZVL: Zoonotic visceral leishmaniasis
