# Supplementary figures and images for: Phlebotomine mortality effect of systemic insecticides administered to dogs
Source: Parasit Vectors. 2018 Apr 5;11:230. doi: 10.1186/s13071-018-2820-x (PMC5887228; doi:10.1186/s13071-018-2820-x)

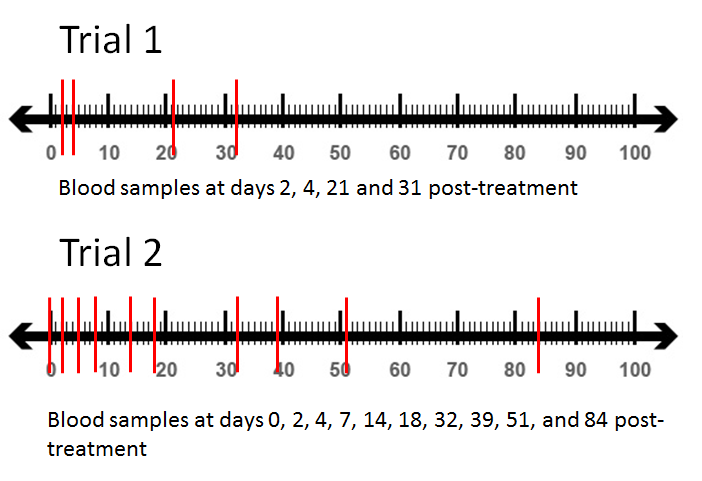

Supplement: Supplementary file 1 — Figure S1. Organization chart showing the sampling schedule. (TIFF 108 kb) [file 13071_2018_2820_MOESM1_ESM.tif]

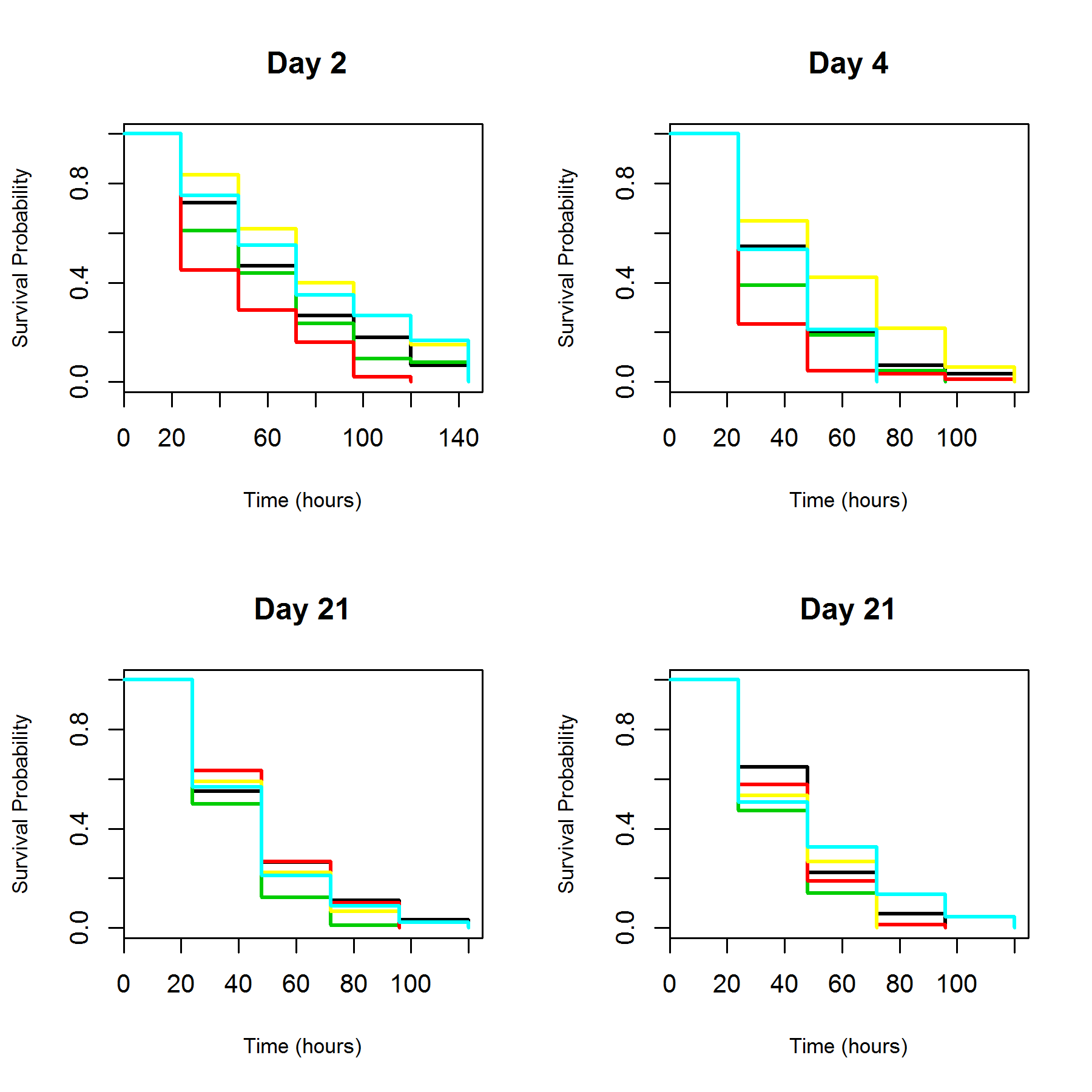

Supplement: Supplementary file 3 — Figure S2. Trial 1, sand fly survival after feeding with blood collected from dogs 2, 4, 21 and 31 days after treatment administration. Groups: control (black); afoxolaner (green); fluralaner (red); moxidectin (yellow); and spinosad (blue). (TIFF 66 kb) [file 13071_2018_2820_MOESM3_ESM.tiff]

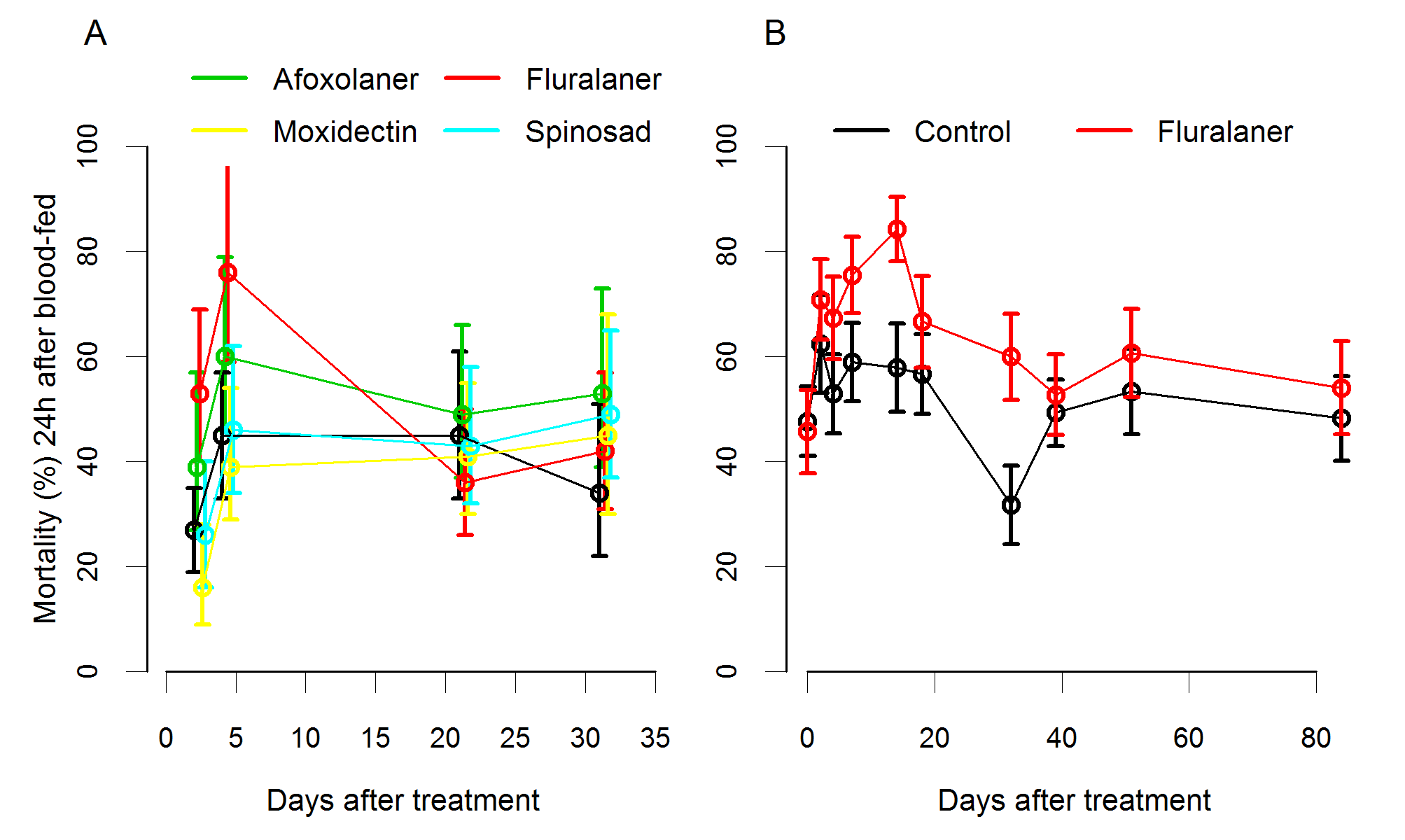

Supplement: Supplementary file 4 — Figure S3. Sand fly mortality percentage and its 95% CI at 24 hours after blood feeding for each treatment group and sampling day. A: Trial 1: afoxolaner vs fluralaner vs moxidectin vs spinosad vs control. B: Trial 2: fluralaner vs control (TIFF 65 kb) [file 13071_2018_2820_MOESM4_ESM.tiff]

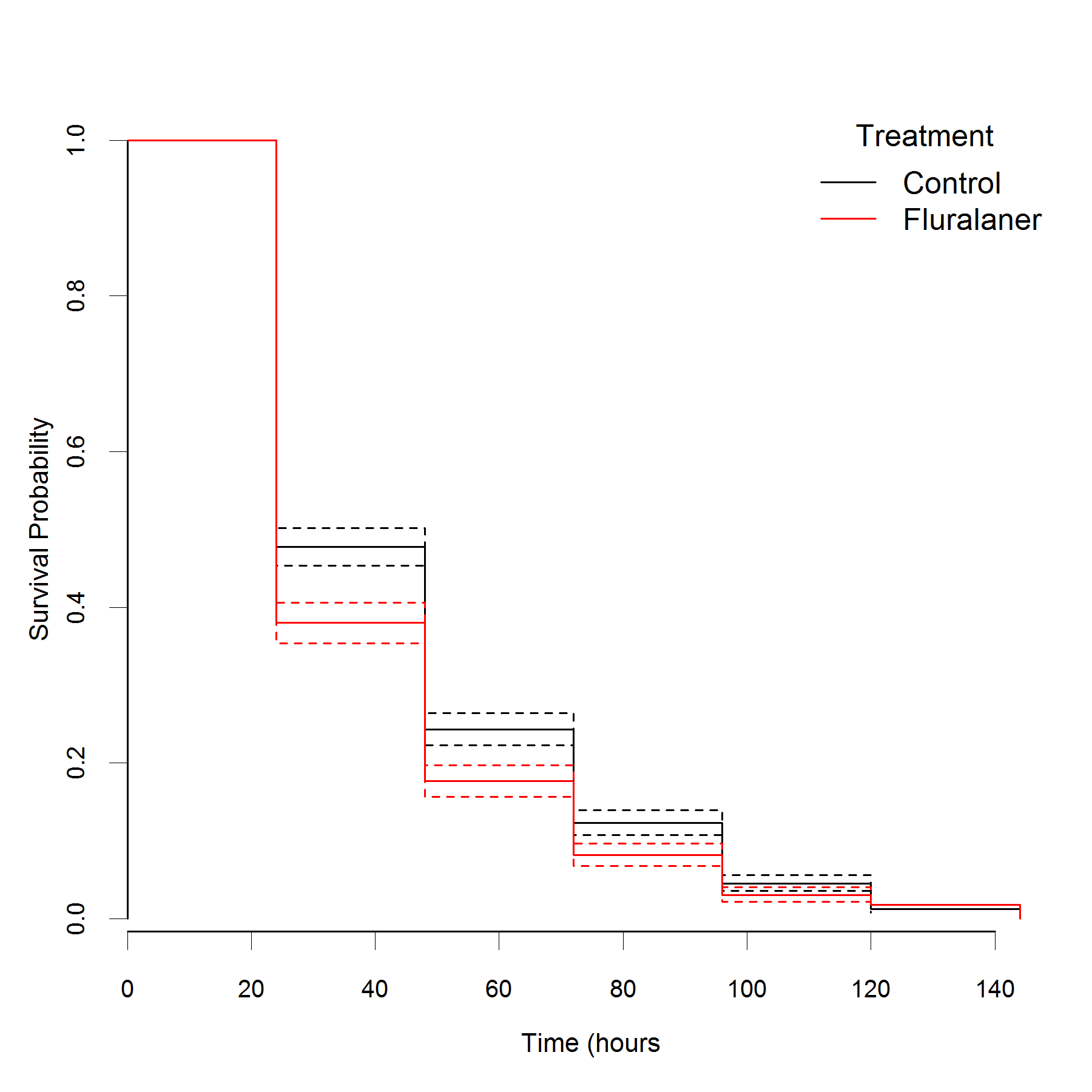

Supplement: Supplementary file 5 — Figure S4. Trial 2, sand fly survival after feeding with blood collected from dogs 0, 2, 4 ,7 14, 18, 32, 39, 51 and 84 days after treatment administration by treatment groups: control (black), fluralaner (red). (TIFF 37 kb) [file 13071_2018_2820_MOESM5_ESM.tiff]
