# Supplementary material for: Phlebotomine mortality effect of systemic insecticides administered to dogs
Source: Parasit Vectors. 2018 Apr 5;11:230. doi: 10.1186/s13071-018-2820-x (PMC5887228; doi:10.1186/s13071-018-2820-x)
Supplement: Supplementary file 2 — Table S1. Randomization results. Table S2. Trial 1, sand fly mortality, percentage, and 95% CI at 24, 48, 72, 96 and 120 hours after blood feeding for treatment group and sampling day. Table S3. Trial 2, sand fly mortality, percentage, and its 95% CI at 24, 48, 72, 96 and 120 hours after blood feeding for treatment group and sampling day. Table S4. Trial 1 results from the negative binomial mixed model with treatment group, sample day and their interaction as explanatory variables and dog as random effect. Table S5. Trial 1 Cox proportional hazard mixed model with treatment group, sample day and their interaction as explanatory variables and dog as random effect. Table S6. Trial 2 negative binomial mixed model with treatment group, sample day and their interaction as explanatory variables and dog as random effect. Table S7. Trial 2 Cox proportional hazard mixed model with treatment group, sample day and their interaction as explanatory variables and dog as random effect (DOCX 53 kb) [file 13071_2018_2820_MOESM2_ESM.docx]

**Additional file 2: Table S1**. Randomization results. F=female, M=male, JR= Jack Russell, PR=Parson Russell, T=Teckel

|  | Trial 1 | | | Trial 2 | | |
| --- | --- | --- | --- | --- | --- | --- |
| Treatment | | Gender (F:M) | Breed (JR:PR:T) | | Gender (F:M) | Breed (JR:PR) |
| Control | | 4:2 | 2:2:2 | | 8:2 | 4:6 |
| Afoxolaner | | 2:4 | 3:1:2 | | -- | -- |
| Fluralaner | | 2:4 | 3:1:2 | | 7:3 | 5:5 |
| Moxidectin | | 4:2 | 3:1:2 | | -- | -- |
| Spinosad | | 4:2 | 2:2:2 | | -- | -- |

**Additional file 2:** **Table S2**. Trial 1, sand fly mortality, percentage, and 95% CI at 24, 48, 72, 96 and 120 hours after blood feeding for treatment group and sampling day

| Treatment | Time |  | Days after treatment | | | | | |  | |  | |
| --- | --- | --- | --- | --- | --- | --- | --- | --- | --- | --- | --- | --- |
|  |  |  | | | Day 2 | Day 4 | Day 21 | | | | Day 31 | |
| Sand flies |  | n | | | 90 | 90 | 90 | | | 90 | |  |
| Control | 24H | ^1^Deaths ^2^(%) | | | 25 (28) | 41 (45) | 41 (45) | | | 32 (35) | |  |
|  |  | ^3^95% CI | | | (19, 38) | (35, 56) | (36, 56) | | | (23, 48) | |  |
|  | 48H | Deaths (%) | | | 23 (53) | 31 (80) | 26 (74) | | | 33 (77) | |  |
|  |  | 95% CI | | | (43,63) | (71, 88) | (65, 83) | | | (66, 88) | |  |
|  | 72H | Deaths (%) | | | 18 (73) | 12 (93) | 14 (90) | | | 13(94) | |  |
|  |  | 95% CI | | | (64, 82) | (88,98) | (83, 96) | | | (88, 100) | |  |
|  | 96H | Deaths (%) | | | 8 (82) | 3 (96) | 7 (97) | | | 3 (100) | |  |
|  |  | 95% CI | | | (74,99) | (92,99) | (94,100) | | | - | |  |
|  | 120H | Deaths (%) | | | 10 (93) | 3 (100) | 2 (100) | | | - | |  |
|  |  | 95% CI | | | (88,99) | - | - | | | - | |  |
| Sand flies |  | n | | | 64 | 90 | 90 | | | 72 | |  |
| Afoxolaner | 24H | Deaths (%) | | | 25 (39) | 55 (61) | 45 (50) | | | 38 (53) | |  |
|  |  | 95% CI | | | (27, 51) | (51, 69) | (40, 60) | | | (42, 64) | |  |
|  | 48H | Deaths (%) | | | 11 (56) | 18 (81) | 34 (87) | | | 26 (88) | |  |
|  |  | 95% CI | | | (44, 51) | (73, 89) | (81, 94) | | | (81, 96) | |  |
|  | 72H | Deaths (%) | | | 13 (76) | 13 (95) | 10 (98) | | | 8 (100) | |  |
|  |  | 95% CI | | | (66, 68) | (91, 99) | (96, 100) | | | - | |  |
|  | 96H | Deaths (%) | | | 9 (90) | 4 (100) | 1 (100) | | | - | |  |
|  |  | 95% CI | | | (83, 87) | - | - | | | - | |  |
|  | 120H | Deaths (%) | | | 1 (92) | - | - | | | - | |  |
|  |  | 95% CI | | | (86, 97) | - | - | | | - | |  |
| Sand flies |  | n | | | 100 | 90 | 90 | | | 85 | |  |
| Fluralaner | 24H | Deaths (%) | | | 55 (55) | 69 (77) | 33 (37) | | | 36 (43) | |  |
|  |  | 95% CI | | | (45, 65) | (68, 86) | (27, 47) | | | (32, 53) | |  |
|  | 48H | Deaths (%) | | | 16 (71) | 17 (95) | 33 (73) | | | 39 (88) | |  |
|  |  | 95% CI | | | (62, 80) | (91, 99) | (64, 82) | | | (81,95) | |  |
|  | 72H | Deaths (%) | | | 12 (83) | 1 (96) | 15 (90) | | | 9 (98) | |  |
|  |  | 95% CI | | | (75, 90) | (93,100) | (83,96) | | | (96, 100) | |  |
|  | 96H | Deaths (%) | | | 5 (88) | 2 (98) | 9 (100) | | | 1 (100) | |  |
|  |  | 95% CI | | | (81, 94) | (96, 100) | - | | | - | |  |
|  | 120H | Deaths (%) | | | 8 (96) | 1 (100) | - | | | - | |  |
|  |  | 95% CI | | | (92, 99) | - | - | | | - | |  |
| Sand flies |  | n | | | 60 | 90 | 90 | | | 90 | |  |
| Moxidectin | 24H | Deaths (%) | | | 10 (17) | 36 (40) | 37 (42) | | | 41 (46) | |  |
|  |  | 95% CI | | | (8, 27) | (30, 50) | (31, 51) | | | (35, 56) | |  |
|  | 48H | Deaths (%) | | | 13 (38) | 23 (65) | 33 (77) | | | 31 (80) | |  |
|  |  | 95% CI | | | (26, 50) | (55, 75) | (69, 86) | | | (71, 88) | |  |
|  | 72H | Deaths (%) | | | 13 (60) | 21 (89) | 14 (93) | | | 12 (93) | |  |
|  |  | 95% CI | | (47, 72) | | (82, 95) | (88, 98) | | | (88, 98) | |  |
|  | 96H | Deaths (%) | | 8 (73) | | 10 (100) | 4 (97) | | | 3 (96) | |  |
|  |  | 95% CI | | (62, 84) | | - | (94,100) | | | (93, 100) | |  |
|  | 120H | Deaths (%) | | 7 (85) | | - | 2 (100) | | | 3 (100) | |  |
|  |  | 95% CI | | (76,94) | | - | - | | | - | |  |
| Sand flies |  | n | | 60 | | 90 | 90 | | | 89 | |  |
| Spinosad | 24H | Death (%) | | 15 (25) | | 42 (47) | 39 (44) | | | 44(50) | |  |
|  |  | 95% CI | | (14, 36) | | (36, 57) | (33, 54) | | | (39, 60) | |  |
|  | 48H | Deaths (%) | | 12 (45) | | 35 (85) | 32 (78) | | | 23 (75) | |  |
|  |  | 95% CI | | (32, 57) | | (78, 93) | (70, 87) | | | (66, 84) | |  |
|  | 72H | Deaths (%) | | 12 (65) | | 13 (100) | 11 (91) | | | 10 (86) | |  |
|  |  | 95% CI | | (53, 77) | | - | (85, 97) | | | (79,93) | |  |
|  | 96H | Deaths (%) | | 5 (73) | | - | 6 (97) | | | 12 (100) | |  |
|  |  | 95% CI | | (62, 84) | | - | (95, 100) | | | - | |  |
|  | 120H | Deaths (%) | | 6 (83) | | - | 2 (100) | | | - | |  |
|  |  | 95% CI | | (74, 92) | | - | | - | | | - |  |

^1^95% CI: 95% Confident Interval for the % of sand fly mortality

^1^Deaths: Number of sand flies found dead at the time of observation

^2^(%) : Percentage of the cumulative mortality observed from the blood feeding bioassay to the time of observation

**Additional file 2:** **Table S3**. Trial 2, sand fly mortality, percentage, and 95% CI at 24, 48, 72, 96 and 120 hours after blood feeding for treatment group and sampling day

| Treatment | Time | |  |  | | Days after treatment | |  | |  | |  | |  | |  | |  | |  |  |  |  |  |
| --- | --- | --- | --- | --- | --- | --- | --- | --- | --- | --- | --- | --- | --- | --- | --- | --- | --- | --- | --- | --- | --- | --- | --- | --- |
|  |  | |  |  | | Day 0 | Day 2 | | Day 4 | | Day 7 | | Day 14 | | Day 18 | | Day 32 | | Day 39 | | Day 51 | | | Day 84 |
| Sand flies |  | n | | | 220 | | 104 | | 168 | | 166 | | 133 | | 164 | | 166 | | 237 | | 130 | | | 147 |
| Control | 24H | ^1^Deaths ^2^(%) | | | 105 (48) | | 65 (63) | | 89 (53) | | 98 (60) | | 77 (58) | | 93 (57) | | 56 (34) | | 117 (50) | | 69 (53) | | | 71 (49) |
|  |  | ^3^95% CI | | | (41, 54) | | (53, 72) | | (45, 61) | | (52, 67) | | (50, 66) | | (50, 64) | | (27, 41) | | (43, 56) | | (45, 61) | | | (40, 57) |
|  | 48H | Deaths (%) | | | 57 (74) | | 25 (87) | | 37 (75) | | 40 (84) | | 28 (79) | | 31 (76) | | 41 (59) | | 62 (76) | | 35 (80) | | | 39 (75) |
|  |  | 95% CI | | | (68, 80) | | (80, 94) | | (69, 82) | | (78, 89) | | (72, 86) | | (69, 82) | | (51, 64) | | (71, 81) | | (73, 87) | | | (68, 82) |
|  | 72H | Deaths (%) | | | 28 (86) | | 14 (100) | | 30 (93) | | 25 (98) | | 10 (87) | | 11 (82) | | 32 (78) | | 14 (82) | | 9 (86) | | | 17 (87) |
|  |  | 95% CI | | | (82, 90) | | - | | (89, 97) | | (96, 100) | | (81, 93) | | (77, 88) | | (72, 84) | | (77, 87) | | (80, 92) | | | (80, 92) |
|  | 96H | Deaths (%) | | | 29 (99) | | - | | 11 (99) | | 3 (100) | | 5 (90) | | 13 (90) | | 16 (87) | | 27 (93) | | 10 (94) | | | 19 (99) |
|  |  | 95% CI | | | (99, 100) | | - | | (99, 100) | | - | | (85, 95) | | (86, 95) | | (83, 93) | | (90, 96) | | (90, 98) | | | (99, 100) |
|  | 120H | Deaths (%) | | | 1 (100) | |  | | 1 (100) | | - | | 9 (97) | | 11 (97) | | 12 (95) | | 14 (99) | | 3 (97) | | | 1 (100) |
|  |  | 95% CI | | | - | | - | | - | | - | | (94, 99) | | (95, 100) | | (91, 98) | | (99, 100) | | (97, 99) | | | - |
| Sand flies |  | n | | | 153 | | 134 | | 135 | | 135 | | 134 | | 111 | | 136 | | 157 | | 130 | | | 122 |
| Fluralaner | 24H | Deaths (%) | | | 70 (46) | | 95 (71) | | 91 (68) | | 102 (76) | | 113 (85) | | 74 (67) | | 81 (60) | | 64 (42) | | 79 (61) | | | 66 (55) |
|  |  | 95% CI | | | (38, 54) | | (63, 79) | | (60, 75) | | (68, 83) | | (78, 90) | | (58, 76) | | (52, 68) | | (35, 50) | | (53, 69) | | | (45, 63) |
|  | 48H | Deaths (%) | | | 47 (77) | | 21 (87) | | 21 (83) | | 21 (92) | | 17 (97) | | 18 (83) | | 23 (77) | | 52 (74) | | 24 (80) | | | 30 (79) |
|  |  | 95% CI | | | (70, 83)I | | (81, 93) | | (77, 89) | | (87, 96) | | (95, 99) | | (76, 90) | | (70, 84) | | (68, 81) | | (73, 87) | | | (72, 86) |
|  | 72H | Deaths (%) | | | 27 (94) | | 14 (97) | | 15 (94) | | 12 (100) | | 4 (100) | | 8 (90) | | 14 (87) | | 17 (85) | | 9 (86) | | | 11 (88) |
|  |  | 95% CI | | | (90, 98) | | (94, 100) | | (90, 98) | | - | | - | | (85, 95) | | (81, 93) | | (80, 90) | | (80, 92) | | | (82, 94) |
|  | 96H | Deaths (%) | | | 9 (100) | | 4 (100) | | 6 (99) | | - | | - | | 5 (95) | | 8 (93) | | 15 (95) | | 10 (94) | | | 12 (98) |
|  |  | 95% CI | | | - | | - | | (99, 100) | | - | | - | | (90, 99) | | (89, 97) | | (90, 98) | | (90, 98) | | | (95, 100) |
|  | 120H | Deaths (%) | | | - | | - | | 2 (100) | | - | | - | | 2 (97) | | 1 (94) | | 3 (97) | | 3 (97) | | | 3 (100) |
|  |  | 95% CI | | | - | | - | |  | | - | | - | | (93, 100) | | (90, 98) | | (94, 100) | | (97, 99) | | | - |

^1^95% CI: 95% Confident Interval for the % of sand fly mortality

^1^Deaths: Number of sand flies found dead at the time of observation

^2^(%) : Percentage of the cumulative mortality observed from the blood feeding bioassay to the time of observation

**Additional file 2:** **Table S4**. Trial 1 negative binomial mixed model with treatment group, sample day and their interaction as explanatory variables and dog as random effect

| ^1^Fixed effects | | coef | exp(coef) | se(coef) | 95% CI exp(coef) | Pr(>\|z\|) |
| --- | --- | --- | --- | --- | --- | --- |
| Afoxolaner | 0.33 | | 1.39 | 0.32 | (0.74, 2.57) | 0.31 |
| Fluralaner | 0.66 | | 1.94 | 0.28 | (1.11, 3.35) | 0.01* |
| Moxidectin | -0.52 | | 0.60 | 0.40 | (0.27, 1.31) | 0.21 |
| Spinosad | -0.07 | | 0.90 | 0.36 | (0.46, 1.88) | 0.77 |
| Day 4 | 0.49 | | 1.64 | 0.27 | (0.96, 2.77) | 0.07 |
| Day 21 | 0.5 | | 1.64 | 0.27 | (0.97,2.79) | 0.07 |
| Day 31 | 0.2 | | 1.22 | 0.33 | (0.64, 2.33) | 0.54 |
| Afoxolaner x Day 4 | -0.07 | | 0.94 | 0.39 | (0.43, 2.00) | 0.86 |
| Fluralaner x Day 4 | -0.13 | | 0.88 | 0.35 | (0.44, 1.74) | 0.71 |
| Moxidectin x Day 4 | 0.39 | | 1.47 | 0.47 | (0.58, 3.71) | 0.41 |
| Spinosad x Day 4 | 0.1 | | 1.1 | 0.43 | (0.47, 2.56) | 0.82 |
| Afoxolaner x Day 21 | -0.27 | | 0.77 | 0.39 | (0.35,1.63) | 0.50 |
| Fluralaner x Day 21 | -0.88 | | 0.41 | 0.37 | (0.20, 0.85) | 0.01* |
| Moxidectin x Day 21 | 0.42 | | 1.52 | 0.47 | (0.60, 3.82) | 0.37 |
| Spinosad x Day 21 | 0.02 | | 1.02 | 0.43 | (0.44, 2.37) | 0.96 |
| Afoxolaner x Day 31 | 0.11 | | 1.11 | 0.44 | (0.47, 2.64) | 0.80 |
| Fluralaner x Day 31 | -0.43 | | 0.65 | 0.41 | (0.29, 1.45) | 0.29 |
| Moxidectin x Day 31 | 0.81 | | 2.24 | 0.53 | (0.79, 6.35) | 0.13 |
| Spinosad x Day 31 | 0.44 | | 1.56 | 0.46 | (0.63, 3.82) | 0.33 |

^1^Fixed effects: the fixed effects estimates used as baseline comparison control group and day 2 after treatment

**Additional file 2:** **Table S5.** Trial 1 Cox proportional hazard mixed model with treatment group, sample day and their interaction as explanatory variables and dog as random effect

| ^1^Fixed effects | coef | exp(coef) | se(coef) | 95% CI exp(coef) | Pr(>\|z\|) |
| --- | --- | --- | --- | --- | --- |
| Afoxolaner | 0.12 | 1.12 | 0.22 | (0.73, 1.73) | 0.28 |
| Fluralaner | 0.40 | 1.47 | 0.20 | (1.04, 2.21) | 0.01* |
| Moxidectin | -0.31 | 0.73 | 0.22 | (0.47, 1.13) | 0.13 |
| Spinosad | -0.28 | 0.76 | 0.22 | (0.49, 1.63) | 0.28 |
| Day 4 | 0.80 | 2.23 | 0.15 | (1.65, 2.98) | 0.00 |
| Day 21 | 0.64 | 1.90 | 0.15 | (1.41, 2.54) | 0.00 |
| Day 31 | 0.67 | 1.95 | 0.18 | (1.37, 2.78) | 0.00 |
| Afoxolaner x Day 4 | -0.00 | 0.99 | 0.22 | (0.65, 1.54) | 0.99 |
| Fluralaner x Day 4 | 0.06 | 1.06 | 0.21 | (0.70, 1.60) | 0.76 |
| Moxidectin x Day 4 | 0.11 | 1.12 | 0.23 | (0.71, 1.75) | 0.61 |
| Spinosad x Day 4 | 0.39 | 1.48 | 0.23 | (0.94, 2.32) | 0.85 |
| Afoxolaner x Day 21 | 0.13 | 1.14 | 0.22 | (0.74, 1.75) | 0.55 |
| Fluralaner x Day 21 | -0.42 | 0.65 | 0.21 | (0.43, 0.99) | 0.04* |
| Moxidectin x Day 21 | 0.40 | 1.49 | 0.22 | (0.97, 2.29) | 0.07 |
| Spinosad x Day 21 | 0.30 | 1.35 | 0.22 | (0.87, 2.07) | 0.18 |
| Afoxolaner x Day 31 | 0.18 | 1.20 | 0.25 | (0.73, 1.95) | 0.48 |
| Fluralaner x Day 31 | -0.24 | 0.78 | 0.23 | (0.50, 1.23) | 0.31 |
| Moxidectin x Day 31 | 0.39 | 1.48 | 0.27 | (0.87, 2.51) | 0.15 |
| Spinosad x Day 31 | 0.15 | 1.16 | 0.25 | (0.78, 1.72) | 0.55 |

^1^Fixed effects: the fixed effects estimates used as baseline comparison control group and day 2 after treatment

**Additional file 2:** **Table S6**. Trial 2 negative binomial mixed model with treatment group, sample day and their interaction as explanatory variables and dog as random effect

| ^1^Fixed effects | coef | exp(coef) | se(coef) | 95% CI exp(coef) | Pr(>\|z\|) |
| --- | --- | --- | --- | --- | --- |
| Fluralaner | 0.08 | 1.08 | 0.15 | (0.81, 1.45) | 0.57 |
| Daysaftertreat2 | 0.25 | 1.28 | 0.15 | (0.95, 1.72) | 0.09 |
| Daysaftertreat4 | 0.08 | 1.08 | 0.14 | (0.82, 1.42) | 0.53 |
| Daysaftertreat7 | 0.17 | 1.19 | 0.13 | (0.92, 1.53) | 0.19 |
| Daysaftertreat14 | 0.14 | 1.16 | 0.14 | (0.87, 1.51) | 0.30 |
| Daysaftertreat18 | 0.13 | 1.14 | 0.13 | (0.88, 1.47) | 0.33 |
| Daysaftertreat32 | -0.37 | 0.68 | 0.15 | (0.51, 0.92) | 0.01 |
| Daysaftertreat39 | -0.00 | 0.99 | 0.13 | (0.77, 1.29) | 0.99 |
| Daysaftertreat51 | 0.24 | 1.27 | 0.14 | (0.96, 1.67) | 0.08 |
| Daysaftertreat84 | -0.03 | 0.96 | 0.14 | (0.73, 1.27) | 0.78 |
| TreatmentFluralaner:Daysaftertreat2 | 0.21 | 1.24 | 0.21 | (0.81, 1.86) | 0.35 |
| TreatmentFluralaner:Daysaftertreat4 | 0.33 | 1.40 | 0.20 | (0.94, 2.06) | 0.13 |
| TreatmentFluralaner:Daysaftertreat7 | 0.35 | 1.42 | 0.20 | (0.96, 2.10) | 0.11 |
| TreatmentFluralaner:Daysaftertreat14 | 0.49 | 1.64 | 0.20 | (1.10, 2.41) | 0.02* |
| TreatmentFluralaner:Daysaftertreat18 | 0.19 | 1.22 | 0.21 | (0.80, 1.82) | 0.38 |
| TreatmentFluralaner:Daysaftertreat32 | 0.68 | 1.97 | 0.21 | (1.31, 2.98) | 0.00* |
| TreatmentFluralaner:Daysaftertreat39 | -0.09 | 0.92 | 0.21 | (0.60, 1.38) | 0.73 |
| TreatmentFluralaner:Daysaftertreat51 | 0.21 | 1.24 | 0.21 | (0.81, 1.86) | 0.14 |
| TreatmentFluralaner:Daysaftertreat84 | -0.08 | 0.93 | 0.22 | (0.59, 1.42) | 0.68 |

^1^Fixed effects: the fixed effects estimates used as baseline comparison control group and day 2 after treatment

**Additional file 2:** **Table S7.** Trial 2 Cox proportional hazard mixed model with treatment group, sample day and their interaction as explanatory variables and dog as random effect

| ^1^Fixed effects | coef | exp(coef) | se(coef) | 95% CI exp(coef) | Pr(>\|z\|) |
| --- | --- | --- | --- | --- | --- |
| TreatmentFluralaner | 0.10 | 1.11 | 0.14 | (0.84, 1.45) | 0.46 |
| Daysaftertreat2 | 0.43 | 1.54 | 0.12 | (1.21, 1.94) | 0.00* |
| Daysaftertreat4 | 0.15 | 1.16 | 0.10 | (0.95, 1.41) | 0.14 |
| Daysaftertreat7 | 0.30 | 1.36 | 0.10 | (1.11, 1.64) | 0.00* |
| Daysaftertreat14 | -0.01 | 0.98 | 0.11 | (0.79, 1.22) | 0.88 |
| Daysaftertreat18 | -0.07 | 0.92 | 0.10 | (0.76, 1.13) | 0.47 |
| Daysaftertreat32 | -0.38 | 0.67 | 0.10 | (0.56, 0.83) | 0.00 |
| Daysaftertreat39 | -0.05 | 0.94 | 0.09 | (0.79, 1.13) | 0.57 |
| Daysaftertreat51 | 0.30 | 1.35 | 0.11 | (1.08, 1.67) | 0.00* |
| Daysaftertreat84 | 0.05 | 1.05 | 0.10 | (0.86, 1.28) | 0.65 |
| TreatmentFluralaner:Daysaftertreat2 | -0.07 | 0.93 | 0.17 | (0.66, 1.30) | 0.89 |
| TreatmentFluralaner:Daysaftertreat4 | 0.14 | 1.15 | 0.16 | (0.84,1.28) | 0.43 |
| TreatmentFluralaner:Daysaftertreat7 | 0.18 | 1.20 | 0.16 | (0.87, 1.64) | 0.12 |
| TreatmentFluralaner:Daysaftertreat14 | 0.59 | 1.81 | 0.16 | (1.32, 2.47) | 0.00* |
| TreatmentFluralaner:Daysaftertreat18 | 0.11 | 1.12 | 0.16 | (0.81, 1.53) | 0.36 |
| TreatmentFluralaner:Daysaftertreat32 | 0.33 | 1.39 | 0.16 | (1.01, 1.90) | 0.09 |
| TreatmentFluralaner:Daysaftertreat39 | -0.15 | 0.85 | 0.15 | (0.64, 1.15) | 0.31 |
| TreatmentFluralaner:Daysaftertreat51 | -0.24 | 0.78 | 0.16 | (0.57, 1.07) | 0.21 |
| TreatmentFluralaner:Daysaftertreat84 | -0.05 | 0.94 | 0.16 | (0.69, 1.30) | 0.09 |

^1^Fixed effects: the fixed effects estimates used as baseline comparison control group and day 2 after treatment
